# Improving hydrocarbon production by engineering cyanobacterial acyl-(acyl carrier protein) reductase

**DOI:** 10.1186/s13068-019-1623-4

**Published:** 2019-12-17

**Authors:** Hisashi Kudo, Yuuki Hayashi, Munehito Arai

**Affiliations:** 1 Department of Life Sciences, Graduate School of Arts and Sciences, The University of Tokyo, 3-8-1 Komaba, Meguro, Tokyo 153-8902 Japan; 2 Department of Physics, Graduate School of Science, The University of Tokyo, 3-8-1 Komaba, Meguro, Tokyo 153-8902 Japan

**Keywords:** Acyl-ACP reductase, Aldehyde-deformylating oxygenase, Cyanobacteria, Hydrocarbon, Protein engineering

## Abstract

**Background:**

Acyl-(acyl carrier protein (ACP)) reductase (AAR) is a key enzyme for hydrocarbon biosynthesis in cyanobacteria, reducing fatty acyl-ACPs to aldehydes, which are then converted into hydrocarbons by aldehyde-deformylating oxygenase (ADO). Previously, we compared AARs from various cyanobacteria and found that hydrocarbon yield in *Escherichia coli* coexpressing AAR and ADO was highest for AAR from *Synechococcus elongatus* PCC 7942 (7942AAR), which has high substrate affinity for 18-carbon fatty acyl-ACP, resulting in production of mainly heptadecene. In contrast, the hydrocarbon yield was lowest for AAR from *Synechococcus* sp. PCC 7336 (7336AAR), which has a high specificity for 16-carbon substrates, leading to production of mainly pentadecane. However, even the most productive AAR (7942AAR) still showed low activity; thus, residues within AAR that are nonconserved, but may still be important in hydrocarbon production need to be identified to engineer enzymes with improved hydrocarbon yields. Moreover, AAR mutants that favor shorter alkane production will be useful for producing diesel fuels with decreased freezing temperatures. Here, we aimed to identify such residues and design a highly productive and specific enzyme for hydrocarbon biosynthesis in *E. coli*.

**Results:**

We introduced single amino acid substitutions into the least productive AAR (7336AAR) to make its amino acid sequence similar to that of the most productive enzyme (7942AAR). From the analysis of 41 mutants, we identified 6 mutations that increased either the activity or amount of soluble AAR, leading to a hydrocarbon yield improvement in *E. coli* coexpressing ADO. Moreover, by combining these mutations, we successfully created 7336AAR mutants with ~ 70-fold increased hydrocarbon production, especially for pentadecane, when compared with that of wild-type 7336AAR. Strikingly, the hydrocarbon yield was higher in the multiple mutants of 7336AAR than in 7942AAR.

**Conclusions:**

We successfully designed AAR mutants that, when coexpressed with ADO in *E. coli*, are more highly effective in hydrocarbon production, especially for pentadecane, than wild-type AARs. Our results provide a series of highly productive AARs with different substrate specificities, enabling the production of a variety of hydrocarbons in *E. coli* that may be used as biofuels.

## Background

Hydrocarbon biosynthesis has gained attention as an alternative to fossil fuels [1–3]. A merit of biofuel production by photosynthetic organisms, including plants and algae, is their carbon-neutral production, which will help to reduce global warming by preventing further accumulation of carbon dioxide in the atmosphere [4–8]. Cyanobacteria are known to produce hydrocarbons of 13–17 carbons in length, which are the main components of diesel fuels [9–15]. Hydrocarbon biosynthesis in cyanobacteria involves two enzymes: acyl-(acyl carrier protein (ACP)) reductase (AAR) and aldehyde-deformylating oxygenase (ADO) [10]. Using NADPH, AAR reduces fatty acyl-ACPs or fatty acyl-coenzyme As (CoAs), which are intermediates of fatty acid metabolism, to fatty aldehydes [10, 16]. Subsequently, ADO converts the fatty aldehydes (C_*n*_) into corresponding hydrocarbons (C_*n*−1_) and formate (HCOOH) [10, 17]. *Escherichia coli* coexpressing cyanobacterial AAR and ADO can produce and secrete hydrocarbons, indicating that AAR and ADO are essential for hydrocarbon biosynthesis [10, 18].

Previously, we analyzed the amount of hydrocarbon produced in *E. coli* coexpressing ADO with AARs derived from eight representative cyanobacteria [19]. We found that the yield of hydrocarbon was highest for the AAR from *Synechococcus elongatus* PCC 7942 (7942AAR) and lowest for the AAR from *Synechococcus* sp. PCC 7336 (7336AAR). The hydrocarbon yield was dependent on both the activity and amount of soluble AAR protein. Our results showed that 7942AAR had the highest activity, while both the activity and amount of the soluble form were low for 7336AAR. However, even the activity of 7942AAR was low, with a turnover rate of 0.51 min^−1^ [20]. Thus, increasing both the activity and amount of soluble AAR will be essential for improving hydrocarbon yield. By comparing the amino acid sequences of the AARs that produced the highest and lowest levels of hydrocarbons, i.e., 7942AAR and 7336AAR, respectively, it will be possible to identify nonconserved residues that are likely to be essential for improving hydrocarbon production [21].

Interestingly, the substrate specificity of AAR depends on the habitats of the derived cyanobacteria [19]. We showed that 7942AAR derived from a freshwater cyanobacterium had a high substrate affinity for 18-carbon fatty acyl-ACP/CoA, while 7336AAR derived from a marine cyanobacterium had a high substrate affinity for 16-carbon fatty acyl-ACP/CoA [19]. Thus, *E. coli* coexpressing ADO with either 7942AAR or 7336AAR produced mainly heptadecene (C17:1) or pentadecane (C15:0), respectively. Because shorter alkanes have lower freezing temperatures and because alkanes have higher cetane numbers than alkenes [22, 23], AAR mutants that favor the production of shorter alkanes may be useful for producing diesel fuels with decreased freezing temperatures. Moreover, the construction of a series of highly active AARs with different substrate specificities will provide a means to produce biohydrocarbons of various chain lengths in *E. coli*.

In this study, to identify AAR residues that are essential for improving hydrocarbon production in *E. coli*, we introduced single amino acid substitutions into the least productive AAR (7336AAR) to make its amino acid sequence more similar to that of the most productive AAR (7942AAR). We identified six mutations that are responsible for improving either the activity or amount of soluble AAR, leading to an increase in hydrocarbon yield in *E. coli* coexpressing ADO. Furthermore, by combining these mutations, we succeeded in creating 7336AAR mutants with increased hydrocarbon production, especially for pentadecane, in *E. coli*.

## Results and discussion

### Selection of mutations

**Fig. 1:**
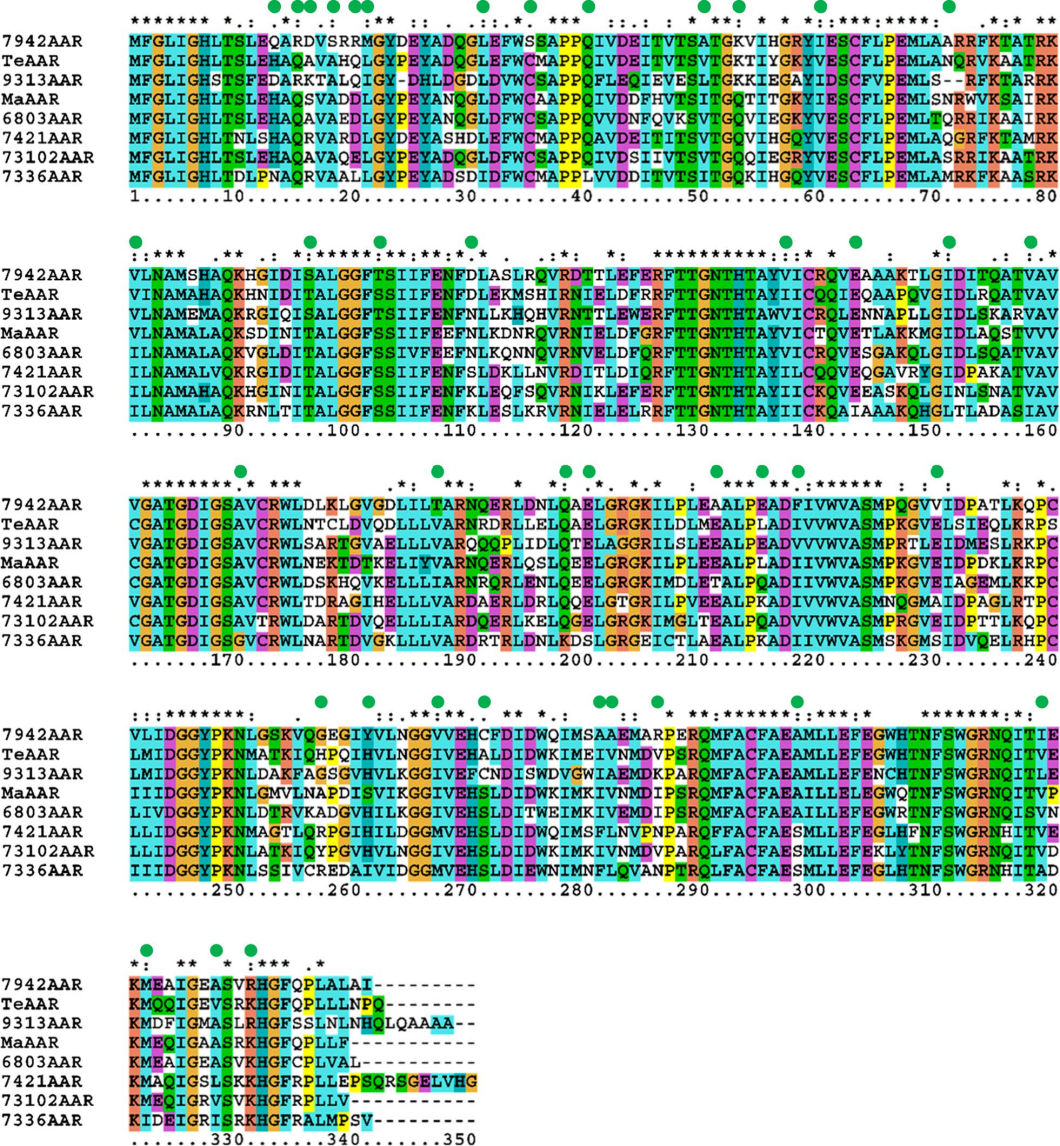
Amino acid sequences of representative AARs [19]. Asterisks denote fully conserved residues, while colons and dots denote partially conserved residues. Green circles highlight the residues of 7336AAR that were mutated in the present study. *Te*AAR, 9313AAR, and *Ma*AAR denote AARs from *Thermosynechococcus elongatus* BP-1, *Prochlorococcus marinus* MIT 9313, and *Microcystis aeruginosa*, respectively

Based on multiple sequence alignment of the AARs (Fig. 1), we designed 41 mutations in 7336AAR that could potentially improve hydrocarbon production when coexpressed with ADO. In Fig. 1, AAR sequences are listed from top to bottom in descending order of hydrocarbon production when coexpressed with ADO in *E. coli* [19]. Hydrocarbon production was highest for 7942AAR and lowest for 7336AAR and AARs from *Nostoc punctiforme* PCC 73102 (73102AAR), *Gloeobacter violaceus* PCC 7421 (7421AAR), and *Synechocystis* sp. PCC 6803 (6803AAR), in ascending order [19].

When the amino acid sequences of the AARs were compared, only nine amino acids of 7336AAR differed from those of other AARs (residues 31, 40, 143, 151, 158, 170, 198, 200, and 322) (Fig. 1). In addition, the amino acid at position 298 was Ser for three AARs that produced low amounts of hydrocarbons (7336AAR, 73102AAR, and 7421AAR), but was Ala for all others. Therefore, the amino acids at these ten positions in 7336AAR may be responsible for its low hydrocarbon yields, and substitution of these amino acids with those of 7942AAR may improve hydrocarbon production. Therefore, we designed ten mutations of 7336AAR, making its amino acid sequence more similar to that of 7942AAR (i.e., I31L, L40Q, I143E, L151I, I158V, G170A, K198Q, S200E, S298A, and I322M).

We also identified the amino acid positions of 7942AAR that differed from the four low hydrocarbon production AARs. Therefore, we selected a further 31 amino acid residues to mutate in 7336AAR, making its amino acid sequence more similar to that of 7942AAR (i.e., N13Q, Q15R, R16D, A18S, L20R, L21M, C35S, I50A, Q53K, V60I, M71A, I81V, T96S, S102T, K110D, I137V, V187T, E211A, K215E, I218F, S230V, R257G, I261Y, M267V, S271C, F281A, L282A, N286R, A319I, I328A, and K331R). In total, 41 mutations were designed, with each mutant 7336AAR having a single amino acid substitution, and their hydrocarbon yields were assessed by a hydrocarbon production assay.

### Hydrocarbon production using 7336AAR mutants

We coexpressed each of the 41 single mutants of 7336AAR with ADO from *Nostoc punctiforme* PCC 73102 (73102ADO) in *E. coli* and attempted to measure the amount of hydrocarbon produced, amount of soluble AAR, AAR activity, and AAR substrate specificity (Additional file 1: Figure S1). 73102ADO was used because it produced the highest hydrocarbon yield when coexpressed with 7942AAR in *E. coli* [10]. Here, the amount of hydrocarbon in the *E. coli* cell culture was measured by gas chromatography–mass spectrometry (GC–MS), while the amount of soluble AAR protein in *E. coli* was quantified by western blotting. AAR activity was calculated as the total amount of hydrocarbon divided by the amount of soluble AAR [19]. Note that in vitro characterization of the catalytic activity of AAR was not possible because AAR proteins are prone to aggregation [16, 19], and we could not obtain 7942AAR in a monomeric form, as judged by gel filtration and small-angle X-ray scattering. Therefore, we previously developed a method for evaluating the in vivo activity of AAR using *E. coli* [19]. The activity obtained by this method is highly reproducible [19, 21].

The results showed that the amount of soluble wild-type 7336AAR was too low to be quantified by western blotting (Additional file 2: Figure S2); therefore, the activity of most mutants and wild-type 7336AAR could not be determined. As 7336AAR was observed in the pellet by western blotting (Additional file 2: Figure S2), we can conclude that wild-type 7336AAR is mostly insoluble. The absence of a soluble enzyme indicates that wild-type 7336AAR has very low solubility.

**Fig. 2:**
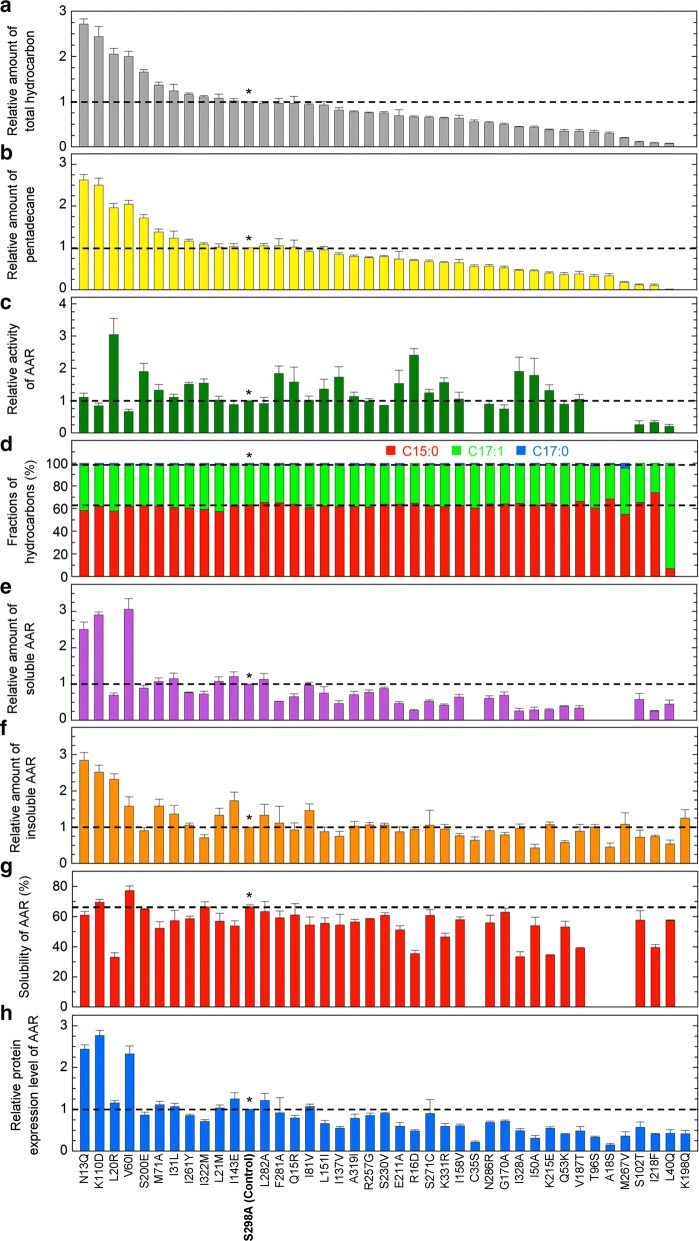
Hydrocarbon production using the 7336AAR double mutants. All mutants contained the S298A mutation in addition to the mutation described at the bottom. **a**, **b** Relative amount of total hydrocarbon (**a**) and pentadecane (**b**) produced in *E. coli* coexpressing 73102ADO and a double mutant of 7336AAR. The data are shown in descending order of the relative amount of total hydrocarbon. **c** Relative activity of AAR. The activities of C35S, T96S, A18S, M267V, and K198Q could not be obtained because the yields of soluble protein were too low to be detected by western blotting. **d** Fractions of pentadecane, heptadecene, and heptadecane relative to the total hydrocarbon yield. **e** Relative amount of soluble AAR in *E. coli*. **f** Relative amount of insoluble AAR in *E. coli*. **g** Solubility of AAR. **h** Relative protein expression levels of AAR. In all panels, a horizontal dotted line shows the value for the S298A single mutant, which was used as a control (denoted by * and “S298A (Control)”). In panels **a**–**c**, **e**, **f**, and **h**, the values are normalized to those of the S298A control. All measurements were taken in triplicate, and the mean ± standard error is shown

Nevertheless, we found that the S298A mutant of 7336AAR was significantly more soluble than the wild-type version, which could be quantified by western blotting (Additional file 2: Figure S2). These results indicate that the S298A mutation greatly improves the solubility of 7336AAR. Thus, to accurately quantify the amount of soluble protein for each of the other 7336AAR mutants, we introduced the S298A mutation into all other single mutants to construct 40 double mutants and measured their hydrocarbon production in *E. coli*. The S298A single mutant was used as a control in the following analysis. Consequently, the amount of hydrocarbon produced in *E. coli* increased for 11 of the 40 double mutants compared with that of the S298A control (Fig. 2a, b). Among them, five double mutants showed enhanced hydrocarbon production by more than 50% (N13Q > K110D > L20R > V60I > S200E; mutations other than S298A are indicated).

The amounts of the soluble and insoluble forms as well as the protein expression levels of AAR in *E. coli* were measured by western blotting. Protein expression was calculated as the total amount of soluble and insoluble AAR. The solubility (%) was calculated as a ratio of the amount of soluble protein to the protein expression level (Fig. 2e–h). An increase in the amount of soluble AAR was observed for 8 of the 40 double mutants compared with that of the S298A control (V60I > K110D > N13Q > I143E > I31L > L282A > L21M > M71A) (Fig. 2e). Seven of these mutants, but not L282A, also showed an increase in the amount of hydrocarbon produced (Fig. 2a). In particular, both the amounts of soluble AAR and hydrocarbon produced increased more than 2.5- and twofold, respectively, for N13Q, V60I, and K110D (Fig. 2a, e). These three mutants were highly soluble, with high levels of protein expression (Fig. 2g, h). The solubility was the highest for V60I (77%).

Improvement in activity was observed for 23 of the 40 double mutants compared with that of the S298A control (Fig. 2c). For the top nine mutants, the activity increased by more than 55% (L20R > R16D > I328A > S200E > F281A > I50A > I137V > Q15R > K331R). Among them, L20R and S200E also showed an increase in hydrocarbon yield; for L20R, the activity and amount of hydrocarbon produced increased more than three- and twofold, respectively (Fig. 2a, c).

*E. coli* coexpressing AAR and ADO produced three types of hydrocarbons, pentadecane (C15:0), heptadecene (C17:1), and heptadecane (C17:0) [10, 19, 21, 24]. When wild-type 7336AAR was used, the major product was pentadecane (Additional file 1: Figure S1c). All of the 7336AAR double mutants except L40Q had the same substrate specificity as the wild-type enzyme (Fig. 2d). For L40Q, the substrate specificity was markedly changed, and the major product was heptadecene; however, both the activity and amount of hydrocarbon produced largely decreased.

### Correlation analysis

**Fig. 3:**
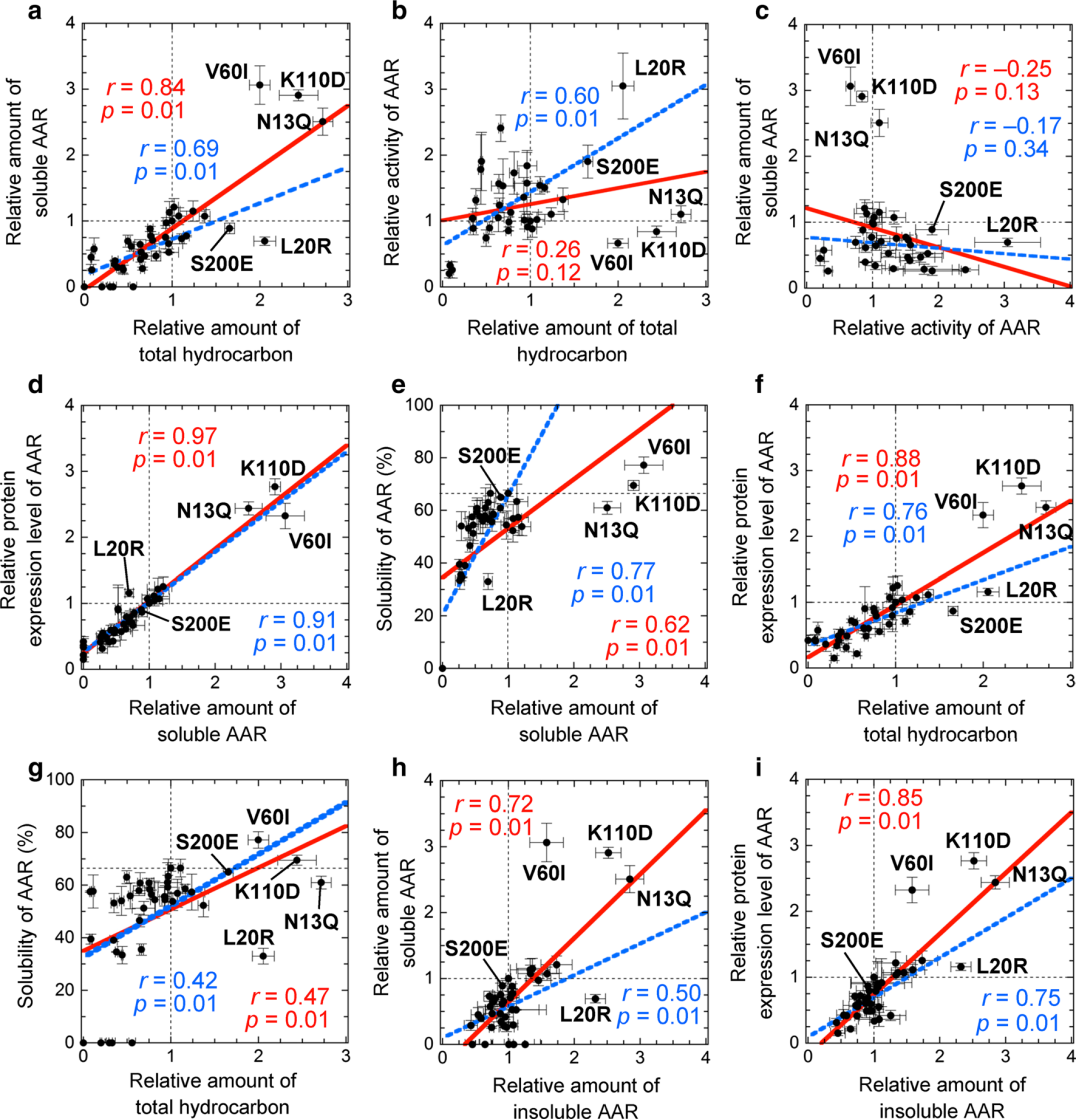
Correlation analysis of the 7336AAR double mutants. **a**, **b** Relative amount of total hydrocarbon plotted against the relative amount of soluble protein (**a**) and relative activity of AAR (**b**). **c** Relative activity of AAR plotted against the relative amount of soluble AAR. **d**, **e** Relative amount of soluble AAR plotted against the relative protein expression level (**d**) and solubility of AAR (**e**). **f**, **g** Relative amount of total hydrocarbon plotted against the relative protein expression level (**f**) and solubility of AAR (**g**). **h**, **i** Relative amount of insoluble AAR plotted against the relative amount of soluble AAR (**h**) and relative protein expression level (**i**). In each panel, a red continuous line indicates a linear regression obtained using all data, and the corresponding correlation coefficient, *r*, and *p* values are shown in red. A blue broken line indicates a linear regression obtained without using the data for N13Q, V60I, and K110D. The data points for the N13Q, L20R, V60I, K110D, and S200E mutants are indicated

Factors directly related to the amount of hydrocarbon produced are the activity and the amount of soluble AAR in the *E. coli* cells. To clarify the degree of contribution of these two factors to hydrocarbon production, we performed a correlation analysis with the double mutant data (Fig. 3). There was a clear correlation between the amount of hydrocarbon produced and the amount of soluble AAR (Fig. 3a). Although the hydrocarbon yield was not significantly correlated with the activity of AAR mutants (Fig. 3b), a positive correlation was observed if data points were omitted for N13Q, V60I, and K110D, which had high levels of soluble AAR. In particular, for L20R and S200E, the increase in hydrocarbon yield was proportional to the increase in activity. These results indicate that both the amount of soluble AAR and activity are important in increasing the amount of hydrocarbon produced. In support of this finding, the activity of AAR was not correlated with the amount of soluble AAR (Fig. 3c); rather, N13Q, V60I, and K110D increased the amount of soluble AAR with little effect on its activity, while L20R and S200E improved the activity almost independent of the amount of soluble AAR.

The amount of soluble AAR and thus the amount of hydrocarbon produced were positively correlated with both the protein expression level and solubility (Fig. 3d–g). Among these variables, the amount of soluble AAR was the most correlated with the protein expression level (correlation coefficient, *r*, of 0.97) (Fig. 3d). Moreover, the amount of insoluble AAR was positively correlated with both the amount of soluble AAR and the protein expression level of AAR (Fig. 3h, i). A notable correlation was not observed for other combinations among the activity, solubility, protein expression level, amount of soluble AAR, amount of insoluble AAR, and amount of hydrocarbon produced (Fig. 3 and Additional file 3: Figure S3).

### Multiple mutations of 7336AAR

**Fig. 4:**
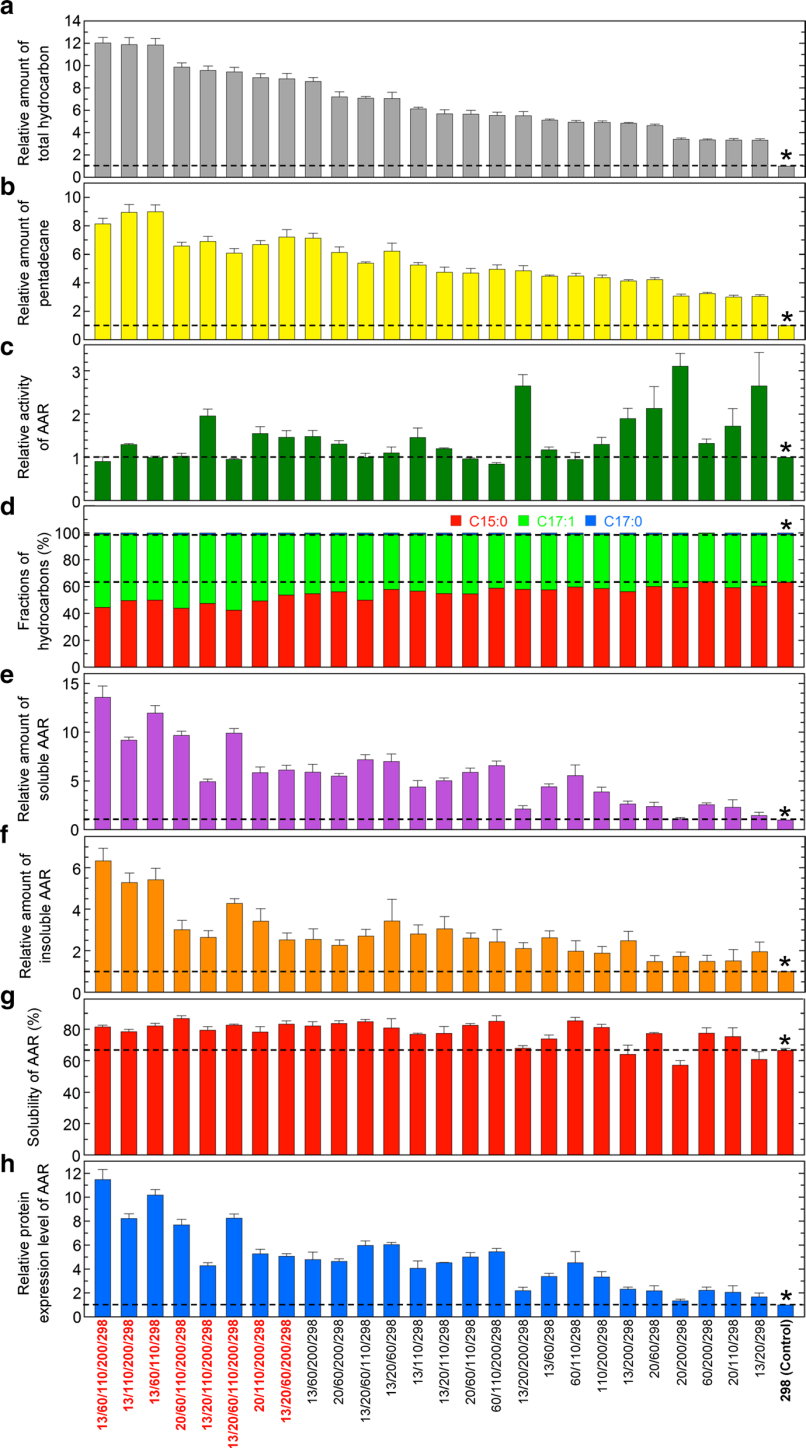
Hydrocarbon production using the 7336AAR multiple mutants. **a**, **b** Relative amount of total hydrocarbon (**a**) and pentadecane (**b**) produced in *E. coli* coexpressing 73102ADO and a multiple mutant of 7336AAR. **c** Relative activity of AAR. **d** Fractions of pentadecane, heptadecene, and heptadecane relative to the total amount of hydrocarbon. **e** Relative amount of soluble AAR in *E. coli*. **f** Relative amount of insoluble AAR in *E. coli*. **g** Solubility of AAR. **h** Relative protein expression level of AAR. In all panels, a horizontal dotted line shows the value for the S298A single mutant, which was used as a control (denoted by * and “298 (Control)”), and 13, 20, 60, 110, 200, and 298 denote the N13Q, L20R, V60I, K110D, S200E, and S298A mutations, respectively. In panels **a**–**c**, **e**, **f**, and **h**, the values are normalized to those of the S298A control. All measurements were taken in triplicate, and the mean ± standard error is shown

The above results showed that the S298A single mutant increased the hydrocarbon yield ~ sixfold compared with that of wild-type 7336AAR (Additional file 1: Figure S1) and that the N13Q, L20R, V60I, K110D, and S200E mutations in combination with S298A further increased the amount of hydrocarbon produced (up to 2.7-fold) compared to that of the S298A control (~ 16-fold greater than that of the wild-type 7336AAR). This result was achieved by either improving the activity (for L20R and S200E) or increasing the amount of soluble AAR (for N13Q, V60I, and K110D) (Fig. 2a, c, e). To produce further increased hydrocarbon yields, we created 26 multiple mutants of 7336AAR by combining the above six mutations. In the following, the N13Q, L20R, V60I, K110D, S200E, and S298A mutations are abbreviated as 13, 20, 60, 110, 200, and 298, respectively. For example, 13/20/298 indicates the 7336AAR mutant with the N13Q, L20R, and S298A mutations. As expected, all multiple mutants increased both the hydrocarbon yield and the amount of soluble AAR and maintained more than 80% activity compared with that of the S298A control (Fig. 4). In particular, three mutants (13/60/110/200/298, 13/110/200/298, and 13/60/110/298) increased the amount of hydrocarbon produced 12-fold compared to that of the S298A control, which corresponds to more than 70-fold increase compared to that of wild-type 7336AAR (Fig. 4a). Two mutants (13/60/110/200/298 and 13/60/110/298) also increased the amount of soluble AAR more than 12-fold compared to that of S298A, while maintaining the activity level (Fig. 4c, e). For 13/110/200/298, both the activity and amount of soluble AAR improved 1.3- and ninefold, respectively, compared to those of S298A (Fig. 4c, e).

Consistent with the fact that the amount of soluble AAR increased with single mutations at the residues 13, 60, 110, and 298 (Fig. 2e; Additional file 1: Figure S1), the multiple mutants that increased the amount of soluble AAR contained mutations of these residues (Fig. 4e). In particular, all four mutations were present in the mutants that improved the amount of soluble AAR more than 12-fold compared to that of S298A (13/60/110/200/298 and 13/60/110/298) (Fig. 4e).

In contrast, substrate specificity and solubility were almost unchanged by the multiple mutations. For most mutants, the major product was pentadecane (Fig. 4d), indicating their higher affinities for 16-carbon fatty acyl-ACP/CoA than for 18-carbon fatty acyl-ACP/CoA substrates. However, as the amount of produced hydrocarbon increased, both the production of longer hydrocarbons (heptadecene) and the solubility of AAR slightly increased (Fig. 4d, g).

**Fig. 5:**
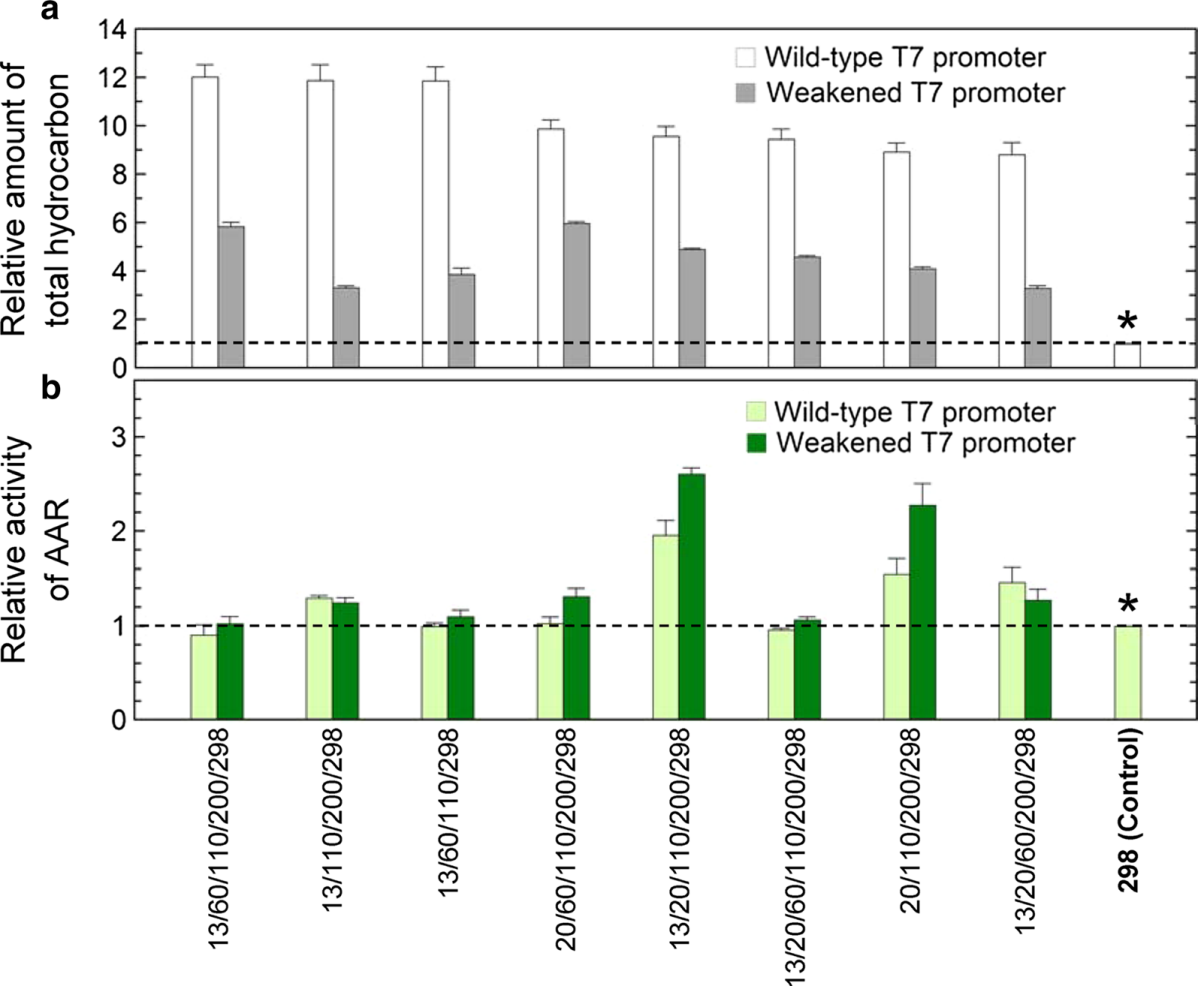
Hydrocarbon production using the 7336AAR multiple mutants with a weakened T7 promoter. **a**, **b** Relative amount of total hydrocarbon (**a**) and relative activity (**b**). Gray (**a**) and dark green bars (**b**) show the relative amount of total hydrocarbon and the relative activity, respectively, obtained using a weakened T7 promoter to decrease protein expression levels. White (**a**) and light green bars (**b**) show the relative amount of total hydrocarbon and the relative activity measured using the wild-type T7 promoter, which are the same as those shown in Fig. 4a, c, respectively. Horizontal dotted lines show the values for the S298A single mutant, which was used as a control (denoted by * and “298 (Control)”)

If AAR is highly active and produces high amounts of aldehydes, conversion of aldehydes into alk(a/e)nes catalyzed by ADO can be a rate-limiting step in the overall process of hydrocarbon biosynthesis in *E. coli*. Thus, when high amounts of hydrocarbons are produced, the activity of AAR may not be accurately measured. To circumvent this possibility, we measured the activities of the top eight multiple mutants of 7336AAR for hydrocarbon production using a weakened T7 promoter to decrease protein expression levels (Fig. 5). We introduced a −13A > G mutation into the T7 promoter upstream of the AAR gene [19]. For all eight mutants, suppression of protein expression decreased hydrocarbon production in *E. coli* (Fig. 5a). These results indicate that suppression of AAR expression prevents the ADO reaction from being the rate-limiting step in the overall process of hydrocarbon biosynthesis, enabling accurate measurement of AAR activity. We found that the activities of 13/20/110/200/298 and 20/110/200/298 were 2.6- and 2.3-fold higher, respectively, than that of the S298A control, although they were 1.9- and 1.5-fold greater, respectively, when protein expression was not suppressed (Figs. 4c, and 5b). The substrate specificity and solubility were almost unchanged by the weakened T7 promoter compared with those obtained using the wild-type T7 promoter (Fig. 4; Additional file 4: Figure S4).

### Comparison of hydrocarbon production with 7942AAR

When wild-type 7336AAR was used with ADO for hydrocarbon production in *E. coli*, the hydrocarbon yield was only ~ 2% that achieved with 7942AAR (Table 1; Additional file 5: Figure S5). Even when the S298A mutant of 7336AAR was used, the yield was ~ 12% of that with 7942AAR. However, multiple mutations in 7336AAR significantly improved hydrocarbon production in *E. coli*, with ~ 1.5-fold increased hydrocarbon yield compared with that for 7942AAR, which corresponds to an ~ 70-fold increase in hydrocarbon production compared with that for wild-type 7336AAR (Table 1; Additional file 5: Figure S5). Moreover, pentadecane production increased ~ twofold using 7336AAR containing multiple mutations compared with that for 7942AAR. Thus, we succeeded in constructing AARs that are more productive than wild-type AARs, i.e., the 13/60/110/200/298, 13/110/200/298, and 13/60/110/298 mutants of 7336AAR.

**Table 1:** Absolute amounts of hydrocarbons produced in *E. coli* coexpressing 73102ADO and AAR using M9 minimal medium

AAR	Mutations	Total hydrocarbons (mg/L)	Pentadecane (C15:0) (mg/L)	Heptadecene (C17:1) (mg/L)	Heptadecane (C17:0) (mg/L)
7336AAR	13/60/110/200/298	14.8 ± 0.9	6.58 ± 0.39	8.06 ± 0.48	0.14 ± 0.01
7336AAR	13/110/200/298	14.6 ± 1.0	7.24 ± 0.49	7.22 ± 0.50	0.15 ± 0.01
7336AAR	13/60/110/298	14.6 ± 0.9	7.27 ± 0.47	7.15 ± 0.47	0.15 ± 0.01
7336AAR	S298A	1.2 ± 0.1	0.78 ± 0.03	0.44 ± 0.02	0.01 ± 0.01
7336AAR	Wild-type	0.2 ± 0.1	0.15 ± 0.01	0.08 ± 0.01	0.00 ± 0.00
7942AAR	Wild-type	10.4 ± 0.4	3.75 ± 0.14	6.48 ± 0.24	0.15 ± 0.01

### Cultivation of *E. coli* using M9-rich medium

In the present study, we used M9 minimal medium for cultivation of *E. coli* to remove background peaks in the GC–MS measurement to allow accurate quantification of produced hydrocarbons. Under these conditions, the hydrocarbon yield for the three most productive multiple mutants of 7336AAR was ~ 15 mg/L of culture (Table 1), although the yield would be increased if we used nutrient-rich media. Thus, we measured hydrocarbon yield in *E. coli* coexpressing ADO and each of the three most productive 7336AAR mutants in “M9-rich” medium [10, 25], which was M9 minimal medium supplemented with casamino acids, vitamins and trace minerals (see section “Methods” for details). The results showed that the total hydrocarbon yield was ~ 30 mg/L in *E. coli* coexpressing ADO with the 13/60/110/200/298, 13/110/200/298, or 13/60/110/298 mutant of 7336AAR (Table 2; Additional file 6: Figure S6). Moreover, ~ 15 mg/L pentadecane was obtained for the 13/60/110/298 mutant. These values are twofold higher than those obtained in *E. coli* grown in M9 minimal medium. Therefore, the use of rich medium improved the hydrocarbon production yield, suggesting that the AAR mutants reported here are useful for producing high amounts of hydrocarbons, especially for pentadecane, in *E. coli* if culture conditions are optimized.

**Table 2:** Absolute amounts of hydrocarbons produced in *E. coli* coexpressing 73102ADO and AAR using M9-rich medium

AAR	Mutations	Total hydrocarbons (mg/L)	Pentadecane (C15:0) (mg/L)	Heptadecene (C17:1) (mg/L)	Heptadecane (C17:0) (mg/L)
7336AAR	13/60/110/200/298	28.0 ± 1.5	11.0 ± 0.5	16.7 ± 1.0	0.28 ± 0.01
7336AAR	13/110/200/298	27.5 ± 1.9	13.1 ± 1.0	14.1 ± 0.9	0.35 ± 0.04
7336AAR	13/60/110/298	29.1 ± 1.5	14.6 ± 0.8	14.2 ± 0.7	0.39 ± 0.02
7336AAR	S298A	6.8 ± 0.3	4.3 ± 0.2	2.3 ± 0.1	0.13 ± 0.01
7942AAR	Wild-type	26.5 ± 0.2	7.7 ± 0.1	18.6 ± 0.1	0.27 ± 0.01

### Mapping the mutation sites onto the domain structure of AAR

**Fig. 6:**
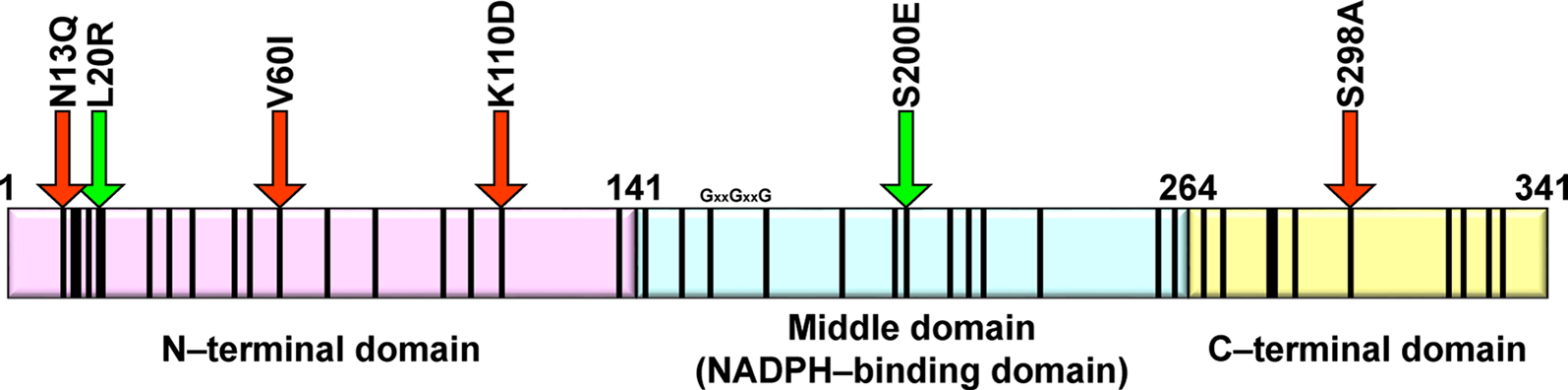
Domain organization of AAR. Mutation sites are shown by black vertical bars. Red and green arrows indicate mutations that increased the amount of soluble AAR or its activity, respectively

The conserved domain search server [26] showed that AAR is composed of at least three domains: an N-terminal domain (residues 1–141), a middle domain (residues 142–264), and a C-terminal domain (residues 265–341) (Fig. 6). In this study, we showed that N13Q, V60I, K110D, and S298A improved the amount of soluble AAR, while L20R and S200E improved its activity (Fig. 2). Three of the mutations that improved the amount of soluble AAR are located in the N-terminal domain, indicating that the N-terminal domain of AAR is essential for protein expression and solubility.

Another mutation that improved the amount of soluble AAR by increasing the solubility of AAR was S298A, which is located in the C-terminal domain of AAR close to the catalytic residue C294 [20]. Secondary structure prediction by the PSIPRED server [27] predicts that residues 289–304 of AAR form an α-helix. Prediction of helical propensity for this region using the AGADIR server [28] indicates that the helical propensity of this region in 7942AAR is ~ threefold higher than that in 7336AAR (Additional file 7: Figure S7). Remarkably, AGADIR predicts that the S298A mutation in 7336AAR will increase the helical propensity of this region by ~ threefold; therefore, the S298A mutant has a helical propensity equivalent to that of 7942AAR (Additional file 7: Figure S7). These results suggest that the S298A mutation stabilizes the α-helix of 7336AAR, and such stabilization of the AAR structure may improve its solubility.

The two mutations that increased enzyme activity were located in the N-terminal and middle domains, suggesting that the N-terminal domain is also important for regulating enzyme activity. The middle domain of AAR contains an NADPH-binding motif (GXXGXXG, where X denotes any amino acid) at residues 162–168 [29, 30]. Thus, the S200E mutation in the middle domain may affect NADPH binding. Detailed mechanisms of how these mutations affect AAR will be elucidated whether and when the three-dimensional structure of AAR is solved.

## Conclusions

In this study, we introduced single amino acid substitutions into the least productive AAR (7336AAR), which has high specificity for 16-carbon fatty acyl-ACP/CoA, to make its amino acid sequence more similar to that of the most productive AAR (7942AAR), which has high affinity for an 18-carbon substrate. By analyzing 41 mutants of 7336AAR, we identified mutations that are responsible for improving either the activity (L20R and S200E) or the amount of soluble AAR (N13Q, V60I, K110D, and S298A), leading to an increase in the amount of hydrocarbon produced, especially for pentadecane, in *E. coli* coexpressing ADO. Moreover, by combining these mutations, hydrocarbon production increased ~ 70-fold compared with that of wild-type 7336AAR, with a hydrocarbon yield higher than that of 7942AAR. Thus, we succeeded in developing AARs that are highly productive (the 13/60/110/200/298, 13/110/200/298, and 13/60/110/298 mutants), even more so than their wild-type counterparts. Our results will provide a series of highly active AARs with different substrate specificities, enabling the production of a variety of hydrocarbons in *E. coli* that can be used as biofuels. The present findings will also be useful for improving the activity of existing enzymes, such as 7942AAR, by introducing various mutations at the six residues identified to determine hydrocarbon yield.

## Methods

### Plasmids

Plasmids containing 7336AAR and 73102ADO were previously constructed based on the pETDuet-1 coexpression vector (Merck Millipore, Darmstadt, Germany) [19]. All AAR and ADO proteins had a C-terminal extension of Gly-Ser-Ser-Gly and a 6 × His-tag. The 7336AAR mutants were constructed using a QuikChange site-directed mutagenesis kit according to the manufacturer’s guidelines (Agilent Technologies, Santa Clara, CA, USA).

### Hydrocarbon production and analysis

Alk(a/e)nes were produced in *E. coli* by coexpression of AAR and ADO as described previously [19, 24, 31]. In brief, *E. coli* BL21 (DE3) pLysS competent cells were transformed with the plasmid described above and inoculated onto a 2 × YT agarose plate containing carbenicillin (50 μg/mL) and chloramphenicol (34 μg/mL). The colonies on the plate were seeded into M9 minimal liquid medium containing ampicillin (50 μg/mL) and chloramphenicol (34 μg/mL) and precultured at 37 °C overnight. The preculture was seeded at an optical density at 600 nm of 0.1 into M9 minimal medium or M9-rich medium containing 100 µM ammonium iron (II) sulfate and 1 mM isopropyl-β-d-1-thiogalactoside (Nacalai Tesque, Kyoto, Japan). M9-rich medium was composed of 6.4 g/L Na_2_HPO_4_, 3 g/L KH_2_PO_4_, 0.5 g/L NaCl, 0.5 g/L NH_4_Cl, 0.5 g/L (NH_4_)_2_SO_4_, 5 g/L casamino acids (BD Biosciences, San Jose, CA, USA), 10 g/L glucose, 0.2 g/L MgCl_2_·6H_2_O, 10 mL/L BME vitamins 100 × solution (Sigma-Aldrich, St. Louis, MO, USA), 10 mL/L trace mineral solution, 50 µg/mL ampicillin, and 34 µg/mL chloramphenicol [25]. The trace mineral solution contained 1.1 g/L CaCl_2_, 2 g/L Na_2_MoO_4_·2H_2_O, 1.9 g/L CuSO_4_·5H_2_O, 0.5 g/L H_3_BO_3_, and 2 g/L ZnCl_2_ with the addition of HCl to maintain the pH at 4.7. The culture was incubated in a 96-deep-well plate at 37 or 30 °C for 16 h. The cell culture was sonicated, and 500 µL of the cell lysate was centrifuged to separate the supernatant and pellet fractions, both of which were used to quantify the amounts of soluble and insoluble AAR and ADO by western blotting. Next, 800 µL of the cell lysate was mixed with an equal amount of ethyl acetate by vortexing. The organic phase was separated from the aqueous phase by centrifugation and was subjected to GC–MS analysis using a Shimadzu gas chromatograph mass spectrometer GCMS-QP2010 Ultra (Shimadzu, Kyoto, Japan) as described previously [19, 21, 24, 31].

AAR activity was calculated as the total amount of hydrocarbons (pentadecane, heptadecene, and heptadecane) produced in the culture divided by the amount of soluble AAR expressed in *E. coli*, which was quantified by western blotting. The measurements were taken three times, and the means ± standard errors are shown.

### Western blotting

The amounts of soluble and insoluble AAR were measured by western blotting as described previously [19]. In brief, supernatant and pellet samples prepared from the same volume of cell lysate were analyzed by sodium dodecyl sulfate–polyacrylamide gel electrophoresis using 12% gels. The proteins were electrotransferred onto a polyvinylidene difluoride membrane (Merck Millipore), and the membrane was blocked with 100 mL of phosphate-buffered saline containing 5% skim milk. The membrane was incubated with an anti-His-tag antibody conjugated with horseradish peroxidase (MBL, Nagoya, Japan). The AAR and ADO proteins, both with a C-terminal His-tag, were visualized by color reactions with 5 mL of 3, 3′, 5, 5′-tetramethylbenzidine solution (ATTO, Tokyo, Japan). Gel images of the western blotting were acquired using a Gel Doc EZ imager (Bio-Rad, Hercules, CA, USA). Quantification of the AAR and ADO bands was performed using ImageLab software (Bio-Rad).

## Supplementary information

**Additional file 1: Figure S1.** Hydrocarbon production using single mutants of 7336AAR. **a**, **b** Relative amount of total hydrocarbon (**a**) and pentadecane (**b**) produced in *E. coli* coexpressing 73102ADO and a single mutant of 7336AAR. The data are shown in descending order of total hydrocarbon yield. **c** Fractions of pentadecane, heptadecene, and heptadecane relative to total hydrocarbon yield. In all panels, a horizontal dotted line shows the value for wild-type 7336AAR, which was used as a control (denoted by * and “WT”). In panels **a** and **b**, the values are normalized to those of the wild-type enzyme. All measurements were taken in triplicate, and the mean ± standard error is shown.

**Additional file 2: Figure S2.** Western blotting of the supernatant and pellet fractions of the *E. coli* cell lysates. The *E. coli* cell culture expressing wild-type (WT) or the S298A mutant of 7336AAR with 73102ADO was sonicated and centrifuged to separate the supernatant (S) and pellet (P) fractions. The bands for 7336AAR (38.8 kDa) and 73102ADO (27.4 kDa) are indicated by arrows. Marker denotes the lane with molecular weight markers (37 and 29 kDa). The lanes labeled pETDuet-1 show the results for *E. coli* transformed with an empty pETDuet-1 plasmid containing neither AAR nor ADO.

**Additional file 3: Figure S3.** Correlation analysis of the 7336AAR double mutants. **a** Relative amount of total hydrocarbon plotted against the relative amount of insoluble AAR. **b, c** Solubility is plotted against the relative amount of insoluble AAR (**b**) and relative protein expression level of AAR (**c**). **d, e, f** Relative activity of AAR plotted against the relative amount of insoluble AAR (**d**), solubility of AAR (**e**), and relative protein expression level of AAR (**f**). In each panel, a red continuous line indicates a linear regression obtained using all data, and the corresponding correlation coefficient, *r*, and *p* values are shown in red. A blue broken line indicates a linear regression obtained without using the data for N13Q, V60I, and K110D. The data points for the N13Q, L20R, V60I, K110D, and S200E mutants are indicated.

**Additional file 4: Figure S4.** Hydrocarbon production using the 7336AAR multiple mutants. A weakened T7 promoter was used to reduce protein expression levels. **a, b** Relative amount of total hydrocarbon (**a**) and pentadecane (**b**) produced in *E. coli* coexpressing 73102ADO and a multiple mutant of 7336AAR. **c** Relative activity of AAR. **d** Fractions of pentadecane, heptadecene, and heptadecane relative to the total amount of hydrocarbon. **e** Relative amount of soluble AAR in *E. coli*. **f** Relative amount of insoluble AAR in *E. coli*. **g** Solubility of AAR. **h** Relative protein expression level of AAR. In all panels, the order of the data is the same as that in Fig. 4. In all panels, a horizontal dotted line shows the value for the S298A single mutant used as a control (denoted by * and “298 (Control)”). In panels **a**-**c**, **e**, **f**, and **h**, the values are normalized to those of the S298A control. All measurements were taken in triplicate, and the mean ± standard error is shown.

**Additional file 5: Figure S5.** Comparison of 7942AAR, wild-type 7336AAR, S298A single mutant, and the three most productive multiple mutants of 7336AAR using M9 minimal medium. **a, b** Absolute amount of total hydrocarbon (**a**) and pentadecane (**b**). **c** Relative activity of AAR. **d** Relative amount of soluble AAR in *E. coli*. In all panels, the values are normalized to those of the S298A single mutant, which was used as a control (denoted by * and “298 (Control)”), and a horizontal dotted line shows the value for the S298A control.

**Additional file 6: Figure S6.** Comparison of 7942AAR, the S298A single mutant, and the three most productive multiple mutants of 7336AAR using M9-rich medium. **a, b** Absolute amount of total hydrocarbon (**a**) and pentadecane (**b**). **c** Relative activity of AAR. **d** Relative amount of soluble AAR in *E. coli*. In all panels, the values are normalized to those of the S298A single mutant, which was used as a control (denoted by * and “298 (Control)”), and a horizontal dotted line shows the value for the S298A control.

**Additional file 7: Figure S7.** Helical propensity of residues 289–304 of AAR predicted by the AGADIR server [28]. Open squares show the results for 7942AAR. Blue and red filled circles show the data for wild-type and the S298A mutant of 7336AAR, respectively.

## Data Availability

The datasets supporting the conclusions of this article are included within the article and its additional files.

## Abbreviations

6803AAR: AAR from *Synechocystis* sp. PCC 6803
73102AAR: AAR from *Nostoc punctiforme* PCC 73102
73102ADO: ADO from *Nostoc punctiforme* PCC 73102
7336AAR: AAR from *Synechococcus* sp. PCC 7336
7421AAR: AAR from *Gloeobacter violaceus* PCC 7421
7942AAR: AAR from *Synechococcus elongatus* PCC 7942
9313AAR: AAR from *Prochlorococcus marinus* MIT 9313
AAR: acyl-ACP reductase
ACP: acyl carrier protein
ADO: aldehyde-deformylating oxygenase
CoA: coenzyme A
GC-MS: gas chromatography–mass spectrometry
*Ma*AAR: AAR from *Microcystis aeruginosa*
*Te*AAR: AAR from *Thermosynechococcus elongatus* BP-1
WT: wild-type
