# Supplementary figures and images for: Improving hydrocarbon production by engineering cyanobacterial acyl-(acyl carrier protein) reductase
Source: Biotechnol Biofuels. 2019 Dec 17;12:291. doi: 10.1186/s13068-019-1623-4 (PMC6916063; doi:10.1186/s13068-019-1623-4)

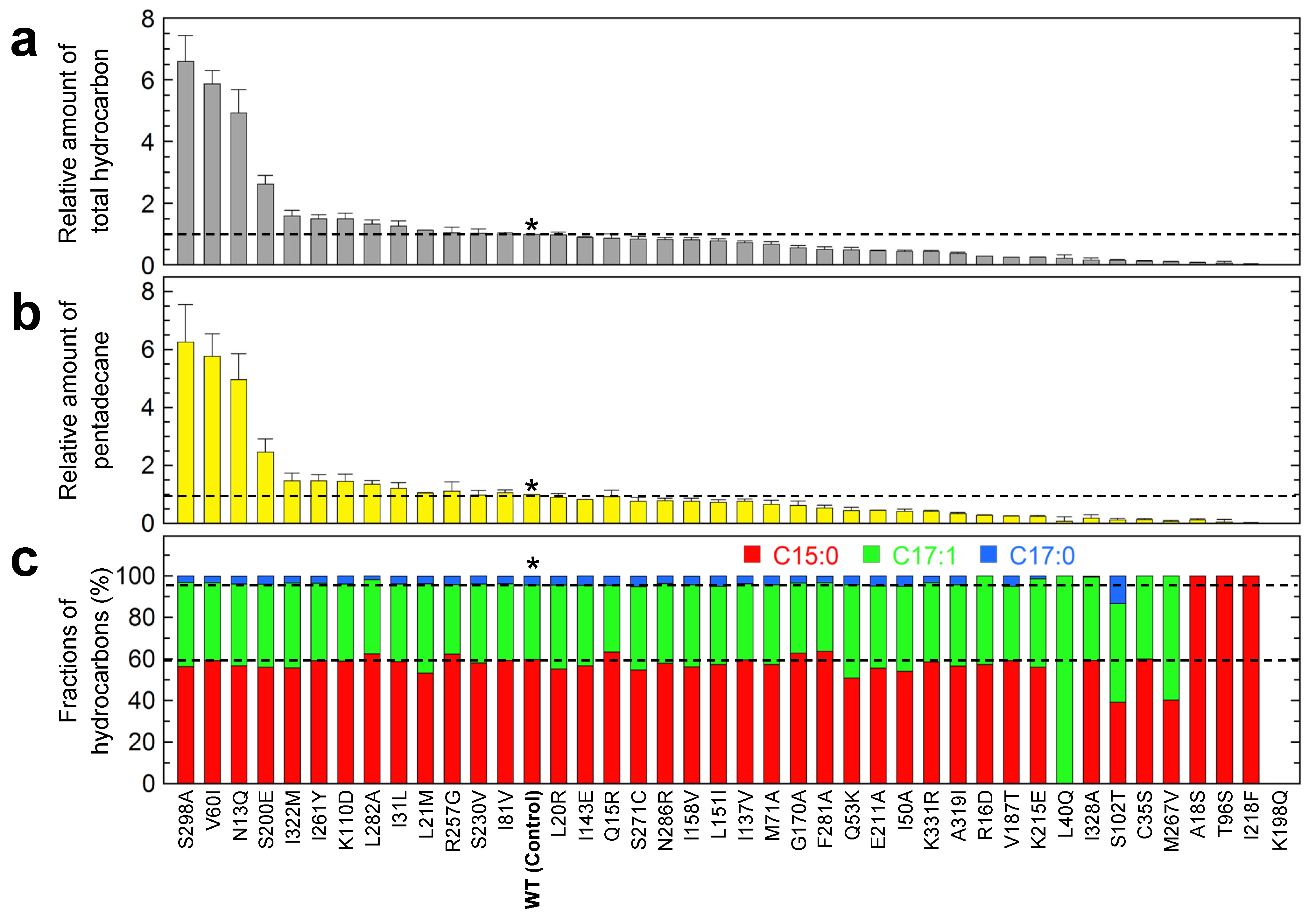

Supplement: Supplementary file 1 — Additional file 1: Figure S1. Hydrocarbon production using single mutants of 7336AAR. a, b Relative amount of total hydrocarbon (a) and pentadecane (b) produced in E. coli coexpressing 73102ADO and a single mutant of 7336AAR. The data are shown in descending order of total hydrocarbon yield. c Fractions of pentadecane, heptadecene, and heptadecane relative to total hydrocarbon yield. In all panels, a horizontal dotted line shows the value for wild-type 7336AAR, which was used as a control (denoted by * and “WT”). In panels a and b, the values are normalized to those of the wild-type enzyme. All measurements were taken in triplicate, and the mean ± standard error is shown. [file 13068_2019_1623_MOESM1_ESM.tif]

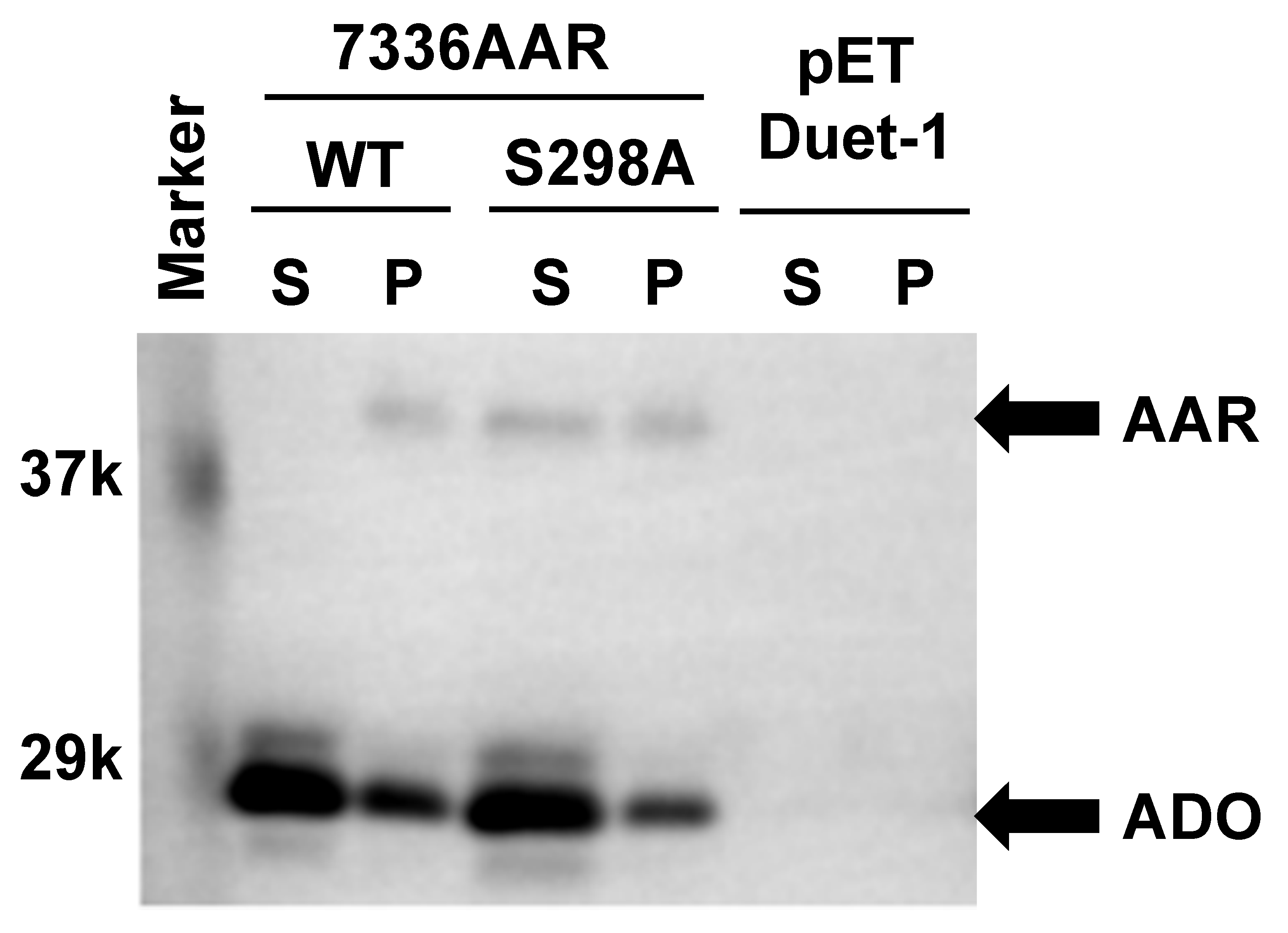

Supplement: Supplementary file 2 — Additional file 2: Figure S2. Western blotting of the supernatant and pellet fractions of the E. coli cell lysates. The E. coli cell culture expressing wild-type (WT) or the S298A mutant of 7336AAR with 73102ADO was sonicated and centrifuged to separate the supernatant (S) and pellet (P) fractions. The bands for 7336AAR (38.8 kDa) and 73102ADO (27.4 kDa) are indicated by arrows. Marker denotes the lane with molecular weight markers (37 and 29 kDa). The lanes labeled pETDuet-1 show the results for E. coli transformed with an empty pETDuet-1 plasmid containing neither AAR nor ADO. [file 13068_2019_1623_MOESM2_ESM.tif]

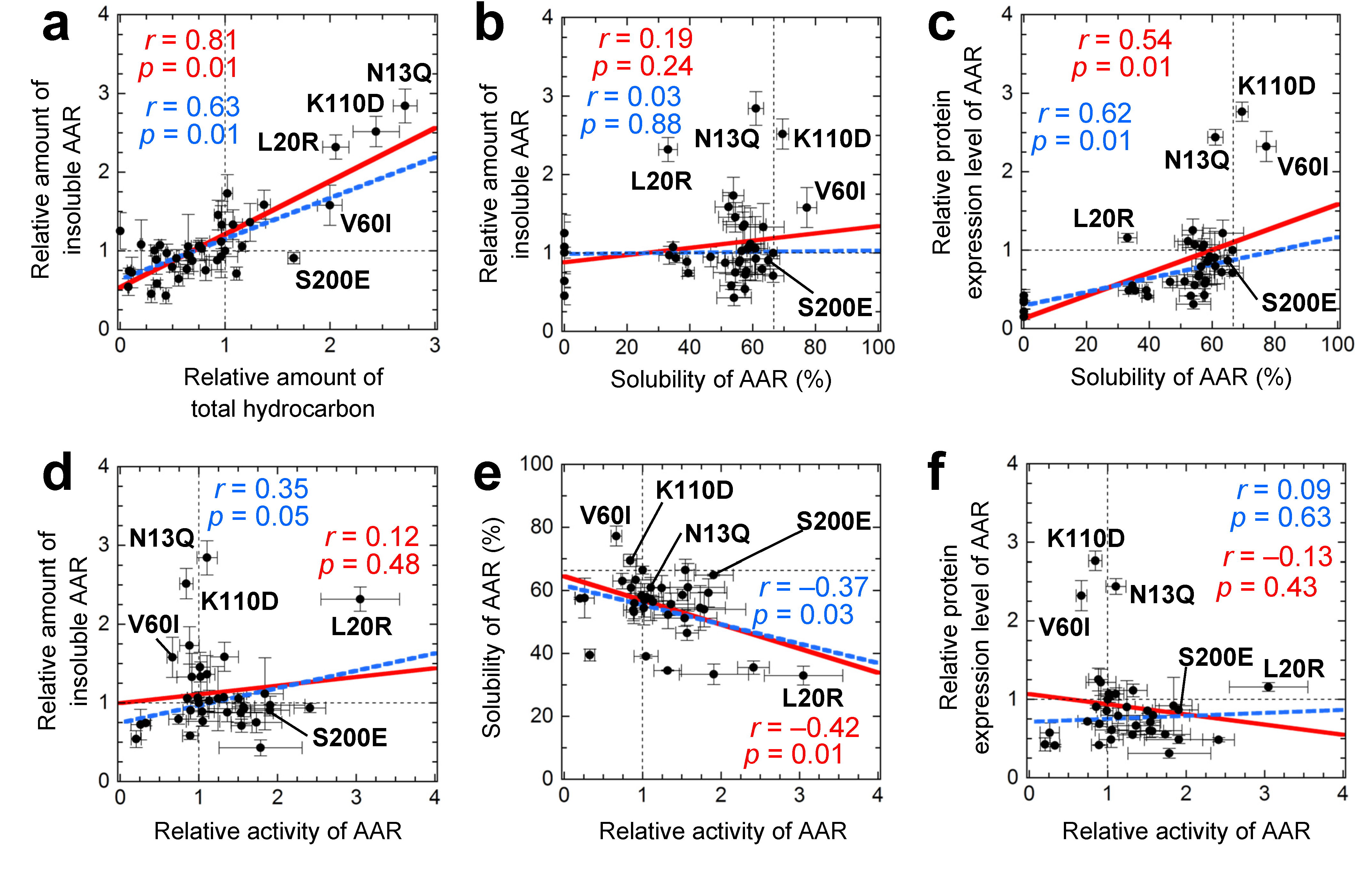

Supplement: Supplementary file 3 — Additional file 3: Figure S3. Correlation analysis of the 7336AAR double mutants. a Relative amount of total hydrocarbon plotted against the relative amount of insoluble AAR. b, c Solubility is plotted against the relative amount of insoluble AAR (b) and relative protein expression level of AAR (c). d, e, f Relative activity of AAR plotted against the relative amount of insoluble AAR (d), solubility of AAR (e), and relative protein expression level of AAR (f). In each panel, a red continuous line indicates a linear regression obtained using all data, and the corresponding correlation coefficient, r, and p values are shown in red. A blue broken line indicates a linear regression obtained without using the data for N13Q, V60I, and K110D. The data points for the N13Q, L20R, V60I, K110D, and S200E mutants are indicated. [file 13068_2019_1623_MOESM3_ESM.tif]

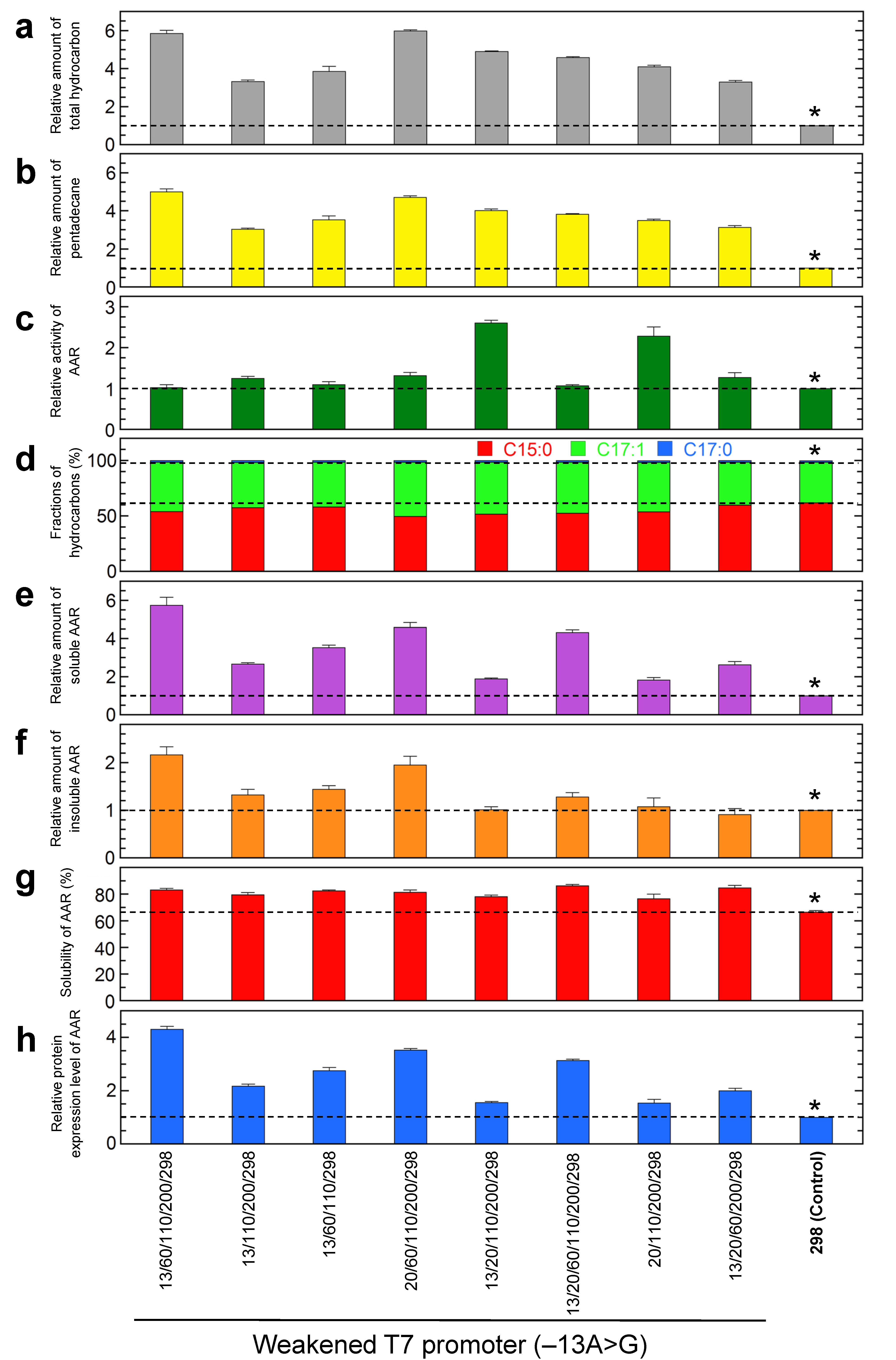

Supplement: Supplementary file 4 — Additional file 4: Figure S4. Hydrocarbon production using the 7336AAR multiple mutants. A weakened T7 promoter was used to reduce protein expression levels. a, b Relative amount of total hydrocarbon (a) and pentadecane (b) produced in E. coli coexpressing 73102ADO and a multiple mutant of 7336AAR. c Relative activity of AAR. d Fractions of pentadecane, heptadecene, and heptadecane relative to the total amount of hydrocarbon. e Relative amount of soluble AAR in E. coli. f Relative amount of insoluble AAR in E. coli. g Solubility of AAR. h Relative protein expression level of AAR. In all panels, the order of the data is the same as that in Fig. 4. In all panels, a horizontal dotted line shows the value for the S298A single mutant used as a control (denoted by * and “298 (Control)”). In panels a-c, e, f, and h, the values are normalized to those of the S298A control. All measurements were taken in triplicate, and the mean ± standard error is shown. [file 13068_2019_1623_MOESM4_ESM.tif]

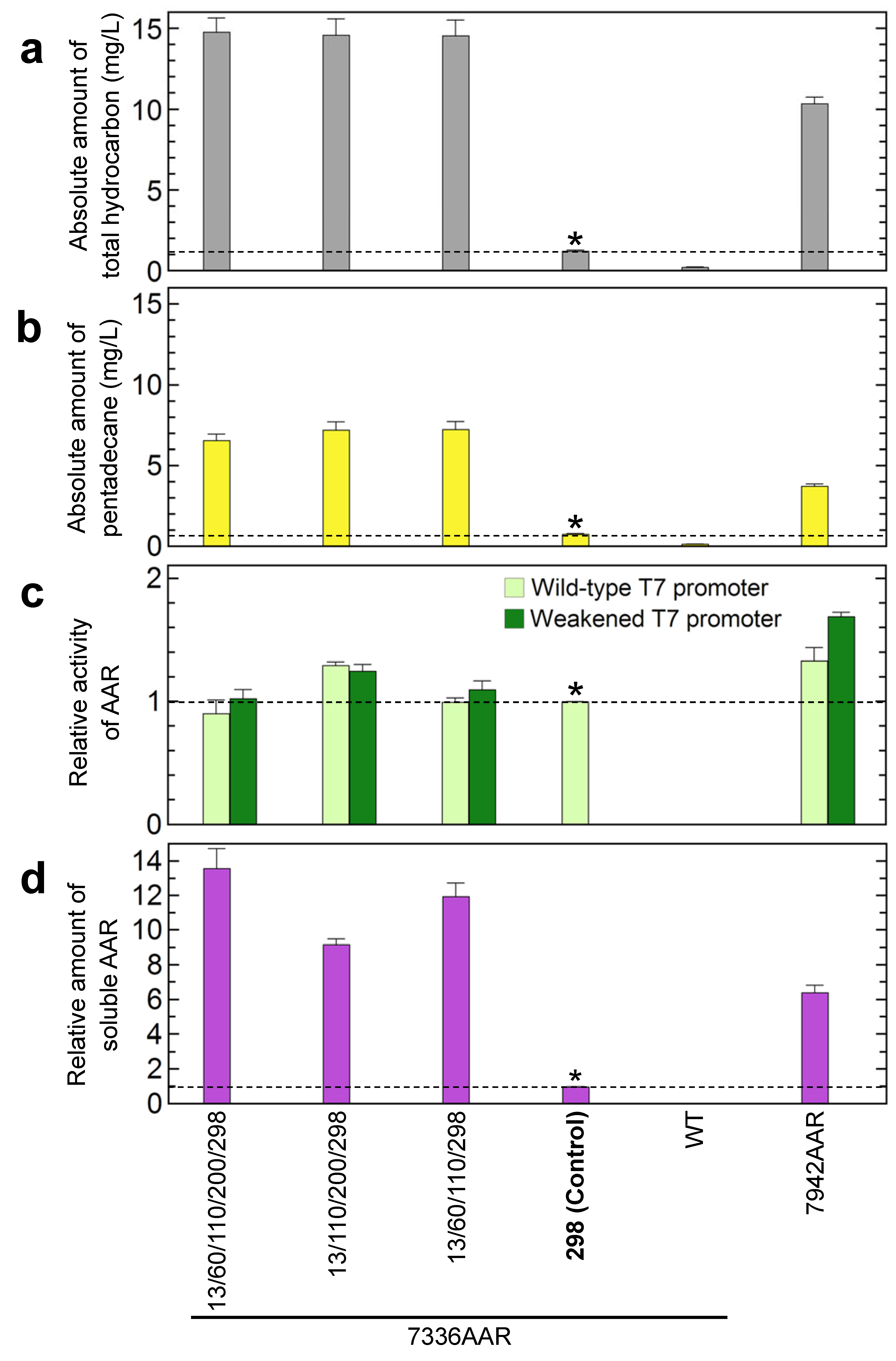

Supplement: Supplementary file 5 — Additional file 5: Figure S5. Comparison of 7942AAR, wild-type 7336AAR, S298A single mutant, and the three most productive multiple mutants of 7336AAR using M9 minimal medium. a, b Absolute amount of total hydrocarbon (a) and pentadecane (b). c Relative activity of AAR. d Relative amount of soluble AAR in E. coli. In all panels, the values are normalized to those of the S298A single mutant, which was used as a control (denoted by * and “298 (Control)”), and a horizontal dotted line shows the value for the S298A control. [file 13068_2019_1623_MOESM5_ESM.tif]

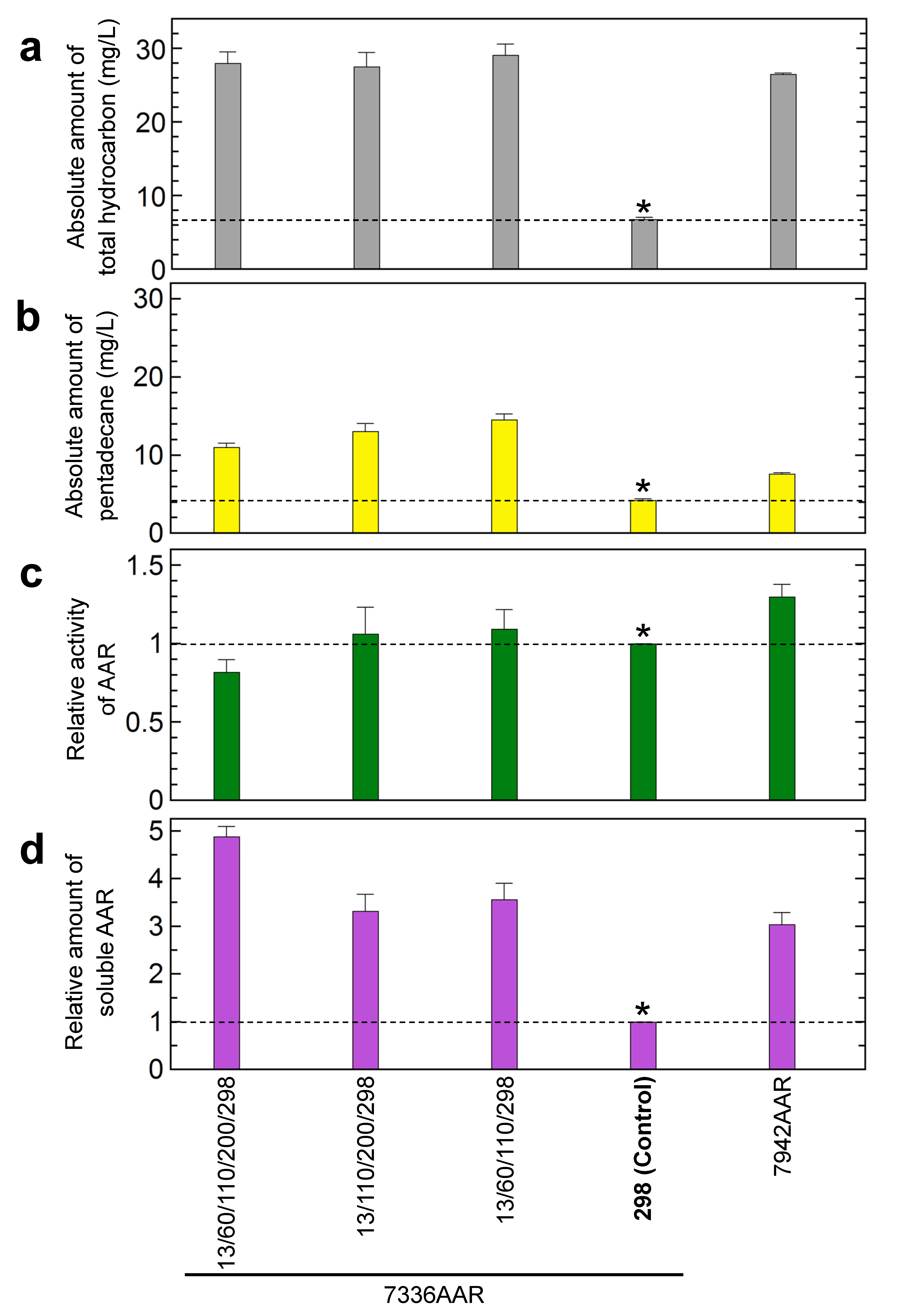

Supplement: Supplementary file 6 — Additional file 6: Figure S6. Comparison of 7942AAR, the S298A single mutant, and the three most productive multiple mutants of 7336AAR using M9-rich medium. a, b Absolute amount of total hydrocarbon (a) and pentadecane (b). c Relative activity of AAR. d Relative amount of soluble AAR in E. coli. In all panels, the values are normalized to those of the S298A single mutant, which was used as a control (denoted by * and “298 (Control)”), and a horizontal dotted line shows the value for the S298A control. [file 13068_2019_1623_MOESM6_ESM.tif]

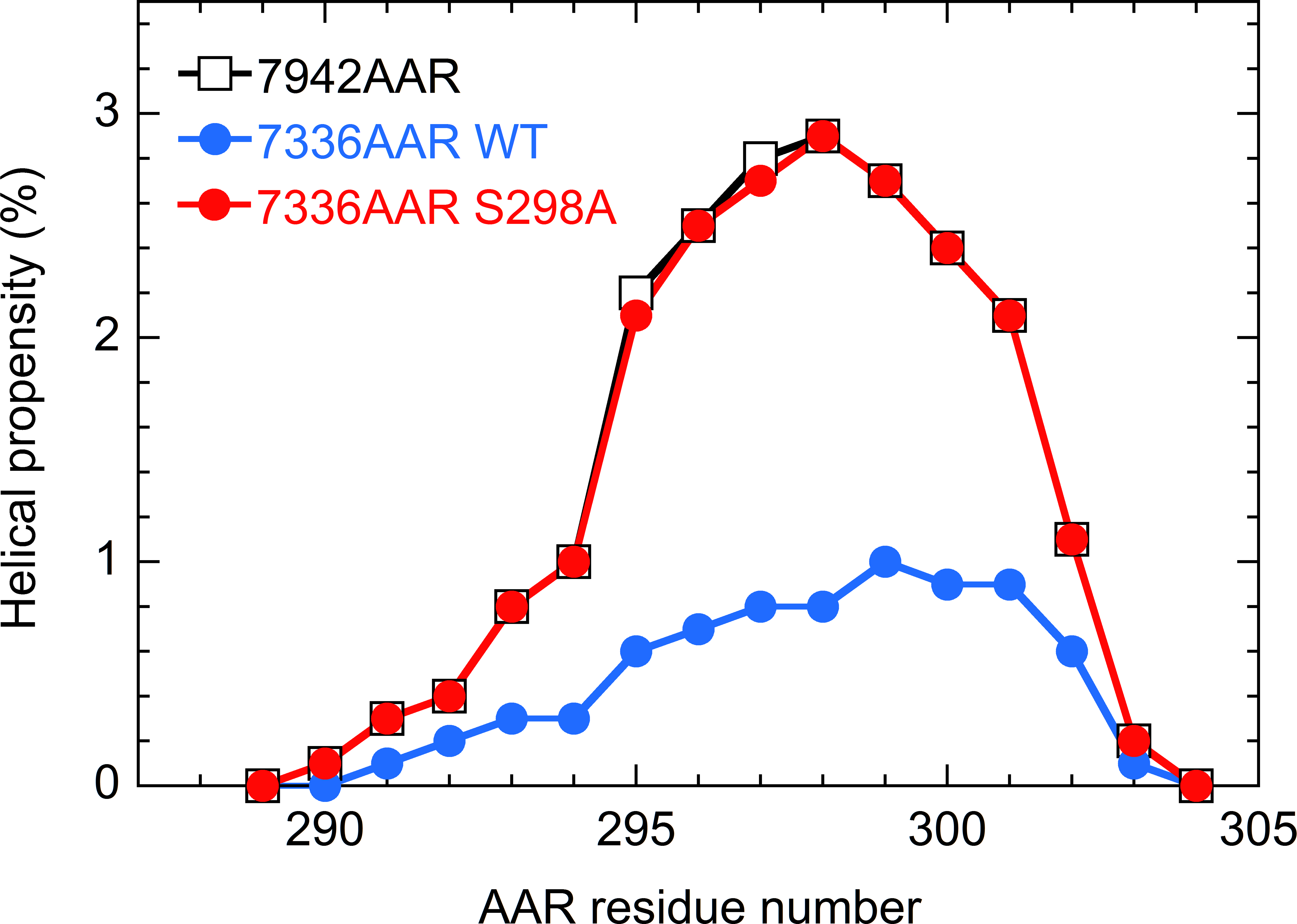

Supplement: Supplementary file 7 — Additional file 7: Figure S7. Helical propensity of residues 289–304 of AAR predicted by the AGADIR server [28]. Open squares show the results for 7942AAR. Blue and red filled circles show the data for wild-type and the S298A mutant of 7336AAR, respectively. [file 13068_2019_1623_MOESM7_ESM.tif]
